# Assessment of miR-1-3p, miR-let-7b-5p, miR-21-5p, and miR-26b-5p in Children with Cardiovascular Diseases

**DOI:** 10.3390/cells15080674

**Published:** 2026-04-10

**Authors:** Marta Pasławska-Zyskowska, Piotr Majewski, Anetta Sulewska, Paweł Muszyński, Miłosz Nesterowicz, Filip Bossowski, Joanna Gościk, Beata Sawicka, Justyna Dunaj-Małyszko, Anna Moniuszko-Malinowska, Jacek Nikliński, Artur Tadeusz Bossowski

**Affiliations:** 1 Department of Pediatrics, Endocrinology and Diabetes with Cardiology Division, Medical University of Bialystok, Jerzego Waszyngtona 17, 15-274 Bialystok, Poland; 2 Department of Microbiological Diagnostics and Infectious Immunology, Medical University of Bialystok, Jerzego Waszyngtona 15A, 15-269 Bialystok, Poland; 3 Department of Clinical Molecular Biology, Medical University of Bialystok, Jerzego Waszyngtona 13, 15-269 Bialystok, Poland; 4 Department of Cardiology, Lipidology and Internal Diseases, Medical University of Bialystok, Żurawia 14, 15-540 Bialystok, Poland; 5 Faculty of Computer Science, Bialystok University of Technology, Wiejska 45A, 15-351 Bialystok, Poland; 6 Department of Infectious Diseases and Neuroinfections, Medical University of Bialystok, Żurawia 14/E, 15-540 Bialystok, Poland

**Keywords:** microRNA, cardiovascular disease, biomarkers, children, arrhythmia, cardiomyopathy, myocarditis, miR-26b-5p, miR-21-5p

## Abstract

Background: Cardiovascular diseases remain important causes of morbidity and potential premature mortality in children. Although clinical imaging and electrophysiologic testing have advanced, early, minimally invasive biomarkers that can both detect myocardial injury and help differentiate among overlapping pediatric phenotypes are still limited. Circulating microRNAs (miRNAs; miRs) are becoming attractive biomarker candidates because many are abundant in the heart, actively released into the circulation, and remarkably stable in plasma. The study aimed to assess the expression of miR-1-3p, miR-let-7b-5p, miR-21-5p, and miR-26b-5p in children with cardiovascular disease. Methods: Children aged 10–18 years with cardiac arrhythmias, myocarditis, or cardio-myopathies were recruited. The control group consisted of healthy age- and sex-matched children. For each participant, peripheral venous blood was collected for plasma isolation and miRNA profiling. The expression of miR-1-3p, miR-let-7b-5p, miR-21-5p, miR-26b-5p, and UniSp6 molecules was analyzed using the comparative cycle threshold delta Ct (ΔCt) method. A *p*-value ≤ 0.05 was considered statistically significant. Results: miR-26b-5p was significantly downregulated in patients with cardiac disease compared with healthy controls. miR-21-5p and miR-26b-5p were downregulated in patients with ventricular arrhythmia. Moreover, miR-26b-5p was downregulated in arrhythmia in general. We found no significant difference in the expression of miR-1-3p, miR-let-7b-5p, miR-21b-5p, and miR-26b-5p between patients with and without myocarditis, as well as with and without hypertrophic cardiomyopathy. Conclusions: miR-26b-5p may distinguish young patients with cardiovascular disease and those with arrhythmias from healthy individuals. miR-21-5p and miR-26b-5p may also be seen as potential biomarkers of ventricular arrhythmia. Further studies involving a larger sample size are required to obtain sufficient data and validate these findings.

## 1. Introduction

Cardiovascular diseases (CVDs) remain important causes of morbidity and potential premature mortality in children, with myocarditis, cardiac arrhythmias, and cardiomyopathies accounting for a substantial proportion of acute presentations and chronic heart failure in this age group. Clinical assessment still relies heavily on imaging, electrocardiography, and non-specific laboratory markers, which can be challenging to interpret in infants and young children and may lack sensitivity for early or subclinical myocardial injury. Conventional blood markers such as troponin and natriuretic peptides are variably elevated, insensitive to low-grade inflammation, and fail to adequately discriminate between arrhythmias, myocarditis, or cardiomyopathy in children. Therefore, recent pediatric-focused reviews and registries highlight the need for blood-based tools that complement standard evaluation in children [1,2,3,4].

Circulating microRNAs (miRNAs; miRs)—short non-coding RNAs that post-transcriptionally regulate gene expression—are attractive biomarker candidates because many are abundant in the heart, actively released into the circulation, and remarkably stable in plasma. Multiple methodological studies demonstrate that, when pre-analytical variables (e.g., hemolysis, processing delays) are controlled, plasma miRNAs show intra-individual stability and disease-associated shifts, supporting their translational promise as clinical biomarkers [5,6,7]. In pediatric cardiology specifically, contemporary syntheses catalog disease-linked miRNAs across arrhythmias, cardiomyopathies, and myocarditis, but also emphasize the scarcity of rigorously designed, cross-condition pediatric studies [8].

In our preliminary study, we wanted to assess the expression of miR-1-3p, miR-let-7b-5p, miR-21-5p, and miR-26b-5p—heart-associated miRNAs previously described in the literature—in children with cardiovascular diseases, as their effects and mechanisms of action are well documented in adults but remain unknown in children.

## 2. Materials and Methods

Children aged 10–18 years were recruited at the Departments of Pediatrics, Endocrinology and Diabetes with Cardiology Division, Medical University of Bialystok. Written informed consent was obtained from parents or legal guardians, and from participants ≥ 16 years of age.

The study group comprised patients with cardiac arrhythmias, myocarditis, or hypertrophic cardiomyopathies. Arrhythmias included supraventricular and ventricular extrasystoles and tachycardias, diagnosed according to established criteria based on symptoms, ECG, and 24 h Holter ECG monitoring. Myocarditis was diagnosed using a composite of clinical presentation (e.g., chest pain, weakness, fever), laboratory abnormalities (elevated blood troponin and creatine kinase), ECG changes (ST-segment elevation/depression, T-wave inversion), rhythm disturbances or conduction abnormalities, PCR identification of a causative pathogen, and multimodal imaging (echocardiography and cardiac MRI). Cardiomyopathies were diagnosed based on ECG, echocardiography, and cardiac MRI.

**Figure 1:**
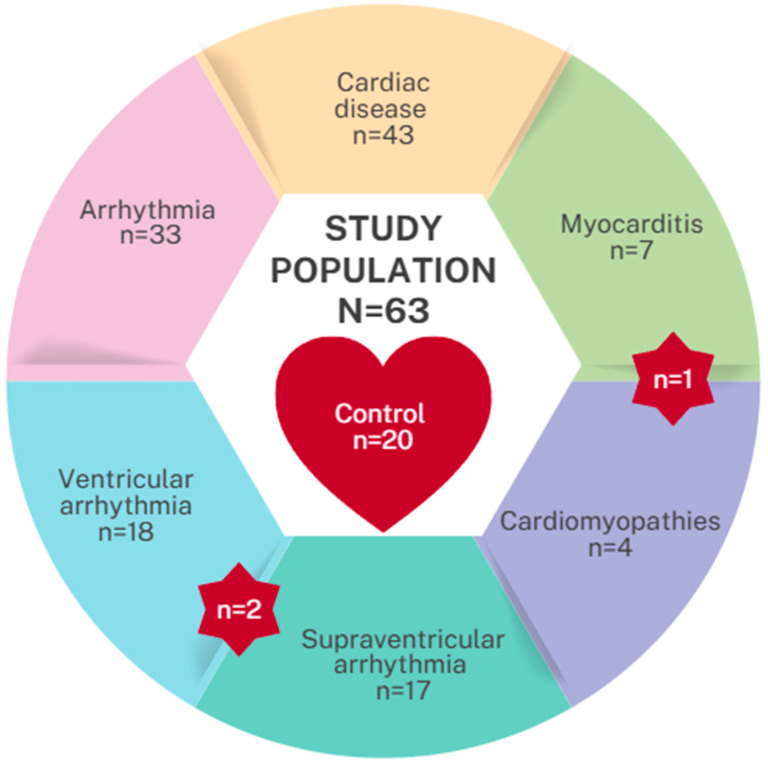
Study group division. Red star highlights patients shared between two groups.

The control group consisted of children without structural heart disease (children referred for evaluation of innocent murmurs, syncope, or chest pain, e.g., Tietze syndrome, neuralgia) and with normal echocardiographic, as well as normal routine laboratory findings. The recruited population consisted of 63 patients, of whom the control group included 20 patients (Figure 1).

Exclusion criteria included coexisting disease (particularly autoimmune disorders such as autoimmune thyroiditis, type 1 diabetes, or juvenile idiopathic arthritis), malignancy, severe systemic illness, known allergies, and lack of written consent; in participants ≥ 16 years of age, absence of personal consent was additionally exclusionary.

For each participant, 3 mL of peripheral venous blood was collected into EDTA tubes (S-Monovette, Sarstedt, Germany) for plasma isolation and miRNA profiling. As soon as the blood was collected, it was frozen at −30 °C.

### 2.1. Plasma Preparation

Defrosted whole blood was centrifuged at 1900× *g* for 10 min at 4 °C. The plasma supernatant was transferred and cleared via a second centrifugation at 16,000× *g* for 10 min at 4 °C. Cleared plasma was immediately aliquoted and stored at −80 °C.

### 2.2. Total RNA Isolation

Circulating total RNA, including miRNA, was isolated from 200 µL of plasma using the miRNeasy Serum/Plasma Advanced Kit (QIAGEN, Hilden, Germany). The protocol followed the manufacturer’s phenol-free, silica-membrane-based purification. RNA was eluted in 14 µL of RNase-free water and stored at −80 °C.

### 2.3. Reverse Transcription and RT-qPCR

Isolated RNA was reverse-transcribed into complementary DNA (cDNA) using the miRCURY LNA RT Kit (QIAGEN, Hilden, Germany). The synthetic UniSp6 Spike-In was added during the reverse transcription reaction and served as an exogenous control to monitor the efficiency of the cDNA synthesis step. The reaction was incubated at 42 °C for 60 min, followed by enzyme inactivation at 95 °C for 5 min. Reverse transcription-quantitative PCR (RT-qPCR) was performed using the miRCURY LNA miRNA PCR Assay system (QIAGEN, Hilden, Germany) on a compatible real-time PCR instrument. Each 10 µL reaction contained the diluted cDNA template and miRCURY LNA SYBR Green Master Mix (QIAGEN, Hilden, Germany). Based on the manufacturer’s validated protocol for locked nucleic acid (LNA)-enhanced primers, amplification was run for an initial polymerase activation step of 95 °C for 2 min, followed by 40 cycles of two-step cycling: 95 °C for 10 s (denaturation) and 56 °C for 60 s (annealing/extension). miRNA expression was quantified using the comparative cycle threshold delta Ct (ΔCt) method, with expression values normalized against the UniSp6 spike-in assay to account for technical variation.

### 2.4. Statistical Analysis

Descriptive statistics, including the mean, standard error of the mean, median, first and third quartiles, as well as the interquartile range, were calculated for grouped data and for data without group affiliation. To determine whether the (selected) variables’ distributions statistically significantly differed between the defined groups, either the *t*-test or its nonparametric equivalent, the two-sample Wilcoxon rank-sum test, was applied. The selection of an appropriate method depended on meeting the assumptions of normality and homogeneity of variances; if any of these assumptions were violated, a non-parametric approach was applied. The normality of features’ distribution was checked with the Shapiro–Wilk test and the homogeneity of variances with the Levene’s test. To assess features’ predictive value, receiver operating characteristic (ROC) curves were generated, optimal cutoff points were determined using the Youden method, and confidence intervals for sensitivity and specificity at the selected threshold were calculated using the Wilson method. In addition, the DeLong test was applied to evaluate whether the area under the curve (AUC) exceeded 0.5 (i.e., random classification); corresponding *p*-values and one-sided confidence intervals for AUC are provided. To account for the potential confounding effects of age and sex, generalized linear models with a logit link function were fitted to assess the effect of miRNA expression levels on the binary outcome (e.g., healthy vs. diseased). All calculations were carried out in the R software environment version 4.5.1 (13 June 2025). An alpha significance level of 0.05 was used for all statistical calculations.

## 3. Results

### 3.1. Characteristics of the Groups

The study population included 63 patients (average age 13.79 years; 57% male). The patients were divided into two groups: healthy control (n = 20) and those with cardiac disease (n = 43), further subdivided into patients with supraventricular arrhythmia (n = 17), ventricular arrhythmia (n = 18), myocarditis (n = 7), and cardiomyopathy (n = 4, all of them were hypertrophic cardiomyopathies [HCM]). Singular patients were included in several groups (two patients had both supraventricular and ventricular arrhythmia, and also, one patient had myocarditis with previous coexisting cardiac hypertrophy). Data were derived from RT-qPCR and presented as delta Ct (ΔCt). Therefore, higher ΔCt values indicate a lower amount of miRNA in the sample. A detailed comparison of the study population is presented in Table 1.

### 3.2. Expression Profiles of miRNAs in the Plasma

**Figure 2:**
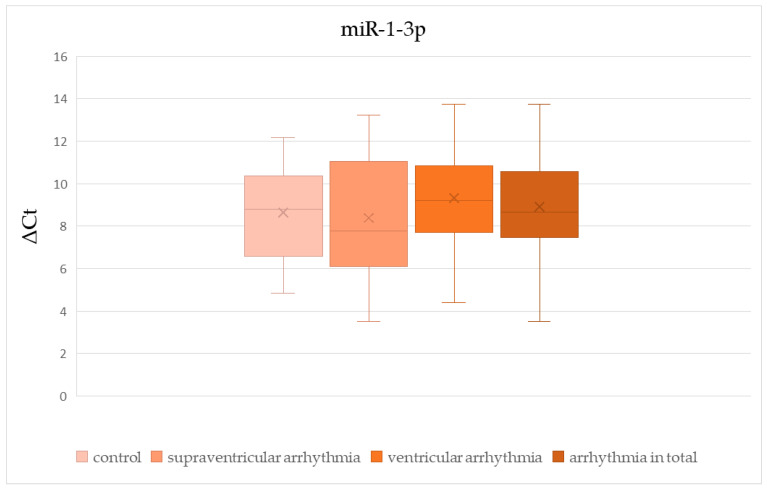
The comparison of miR-1-3p ΔCt values between patients with and without arrhythmias.

**Figure 3:**
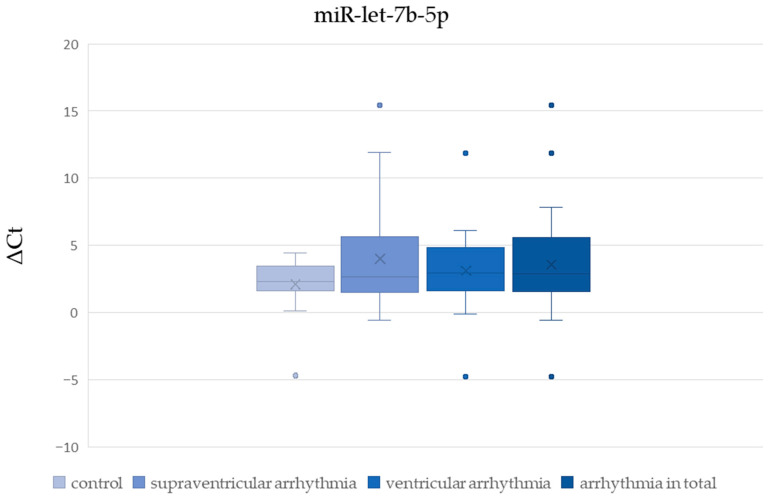
The comparison of miR-let-7b-5p ΔCt values between patients with and without arrhythmias.

**Figure 4:**
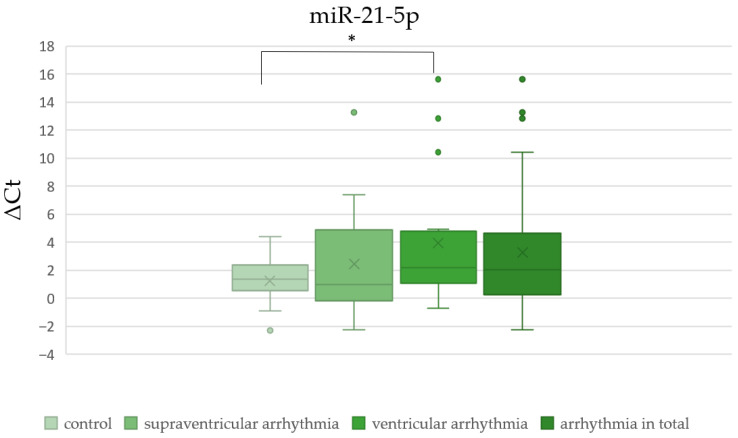
The comparison of miR-21-5p ΔCt values between patients with and without arrhythmias (* *p* < 0.05).

**Figure 5:**
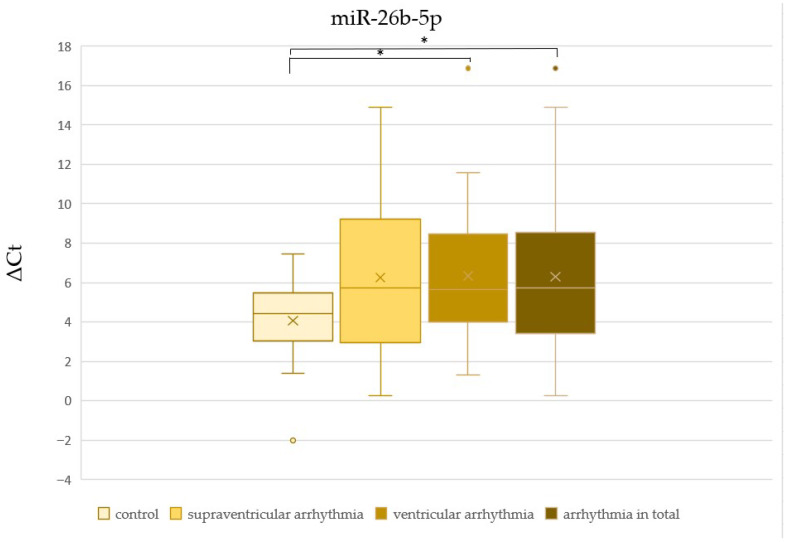
The comparison of miR-26b-5p ΔCt values between patients with and without arrhythmias (* *p* < 0.05).

There was no significant difference in ΔCt values of miR-1-3p, miR-let-7b-5p, and miR-21-5p between patients with and without cardiac disease (Table 2, Figure 2, Figure 3 and Figure 4). The ΔCt value of miR-26b-5p was significantly higher in patients with cardiac disease, indicating downregulation of miR-26b-5p in this group (Table 2, Figure 5).

We found no significant difference in ΔCt values of miR-1-3p, miR-let-7b-5p, miR-21-5p, and miR-26b-5p between patients with supraventricular arrhythmia (Table 3, Figure 2, Figure 3, Figure 4 and Figure 5). On the other hand, ΔCt values of miR-21-5p and miR-26b-5p were significantly higher in patients with ventricular arrhythmia, indicating downregulation of these molecules (Table 4, Figure 4 and Figure 5). The difference in ΔCt values of miR-1-3p and miR-let-7b-5p between patients with and without ventricular arrhythmia has not achieved statistical significance (Table 4, Figure 2 and Figure 3)

There was no significant difference in ΔCt values of miR-1-3p, miR-let-7b-5p, and miR-21-5p between patients with and without arrhythmia in general (Table 5, Figure 2, Figure 3 and Figure 4). ΔCt values of miR-26b-5p were significantly higher in patients with arrhythmias in general compared to healthy controls, indicating downregulation of this miRNA in the whole arrhythmia group (Table 5, Figure 5).

**Figure 6:**
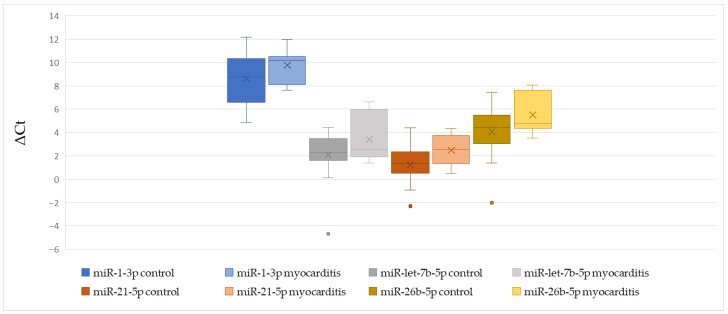
The comparison of miR-1-3p, miR-let-7b-5p, miR-21-5p, and miR-26b-5p ΔCt values between patients with and without myocarditis.

We found no significant difference in ΔCt values of miR-1-3p, miR-let-7b-5p, miR-21-5p, and miR-26b-5p between patients with and without myocarditis (Table 6, Figure 6).

**Figure 7:**
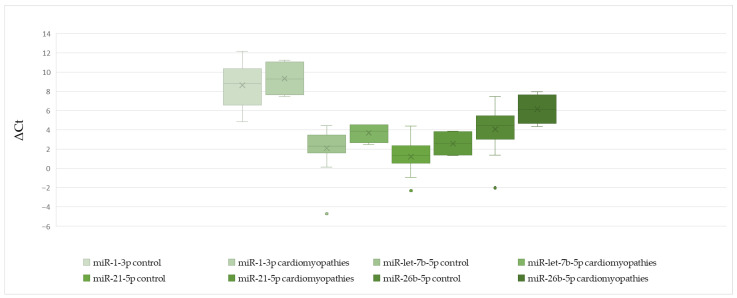
The comparison of miR-1-3p, miR-let-7b-5p, miR-21-5p, and miR-26b-5p ΔCt values between patients with and without cardiomyopathies.

In patients with and without cardiomyopathy, there was no significant difference in miR-1-3p, miR-let-7b-5p, miR-21-5p, and miR-26b-5p (Table 7, Figure 7).

### 3.3. Diagnostic Accuracy

A ROC analysis of miR-21-5p and miR-26b-5p demonstrated that they may have diagnostic potential. Both miRNAs exhibited high specificity; however, they exhibited relatively low sensitivity.

**Figure 8:**
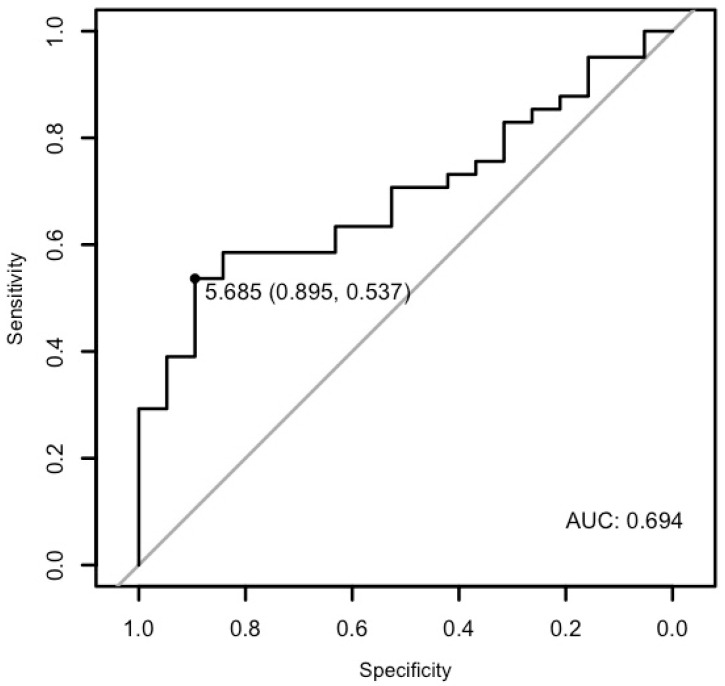
The ROC curve of miR-26b-5p’s predictive value in the research group.

**Figure 9:**
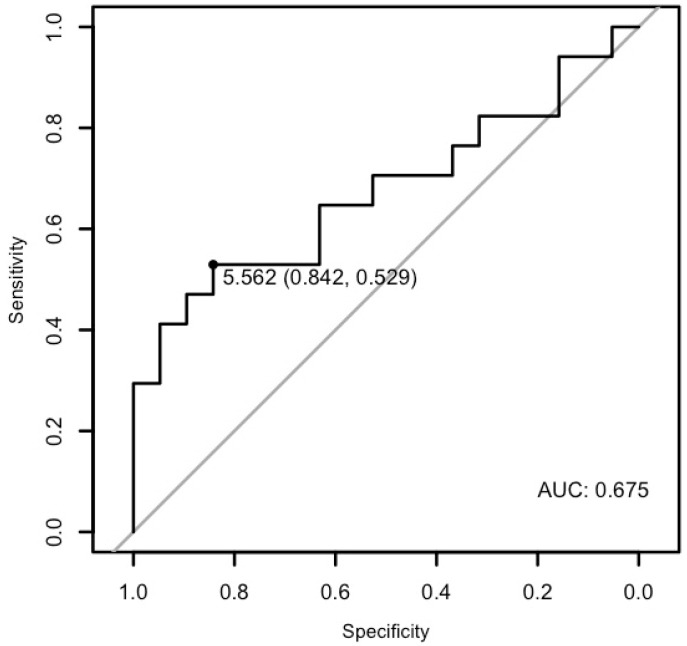
The ROC curve of miR-26b-5p’s predictive value in ventricular arrhythmia.

**Figure 10:**
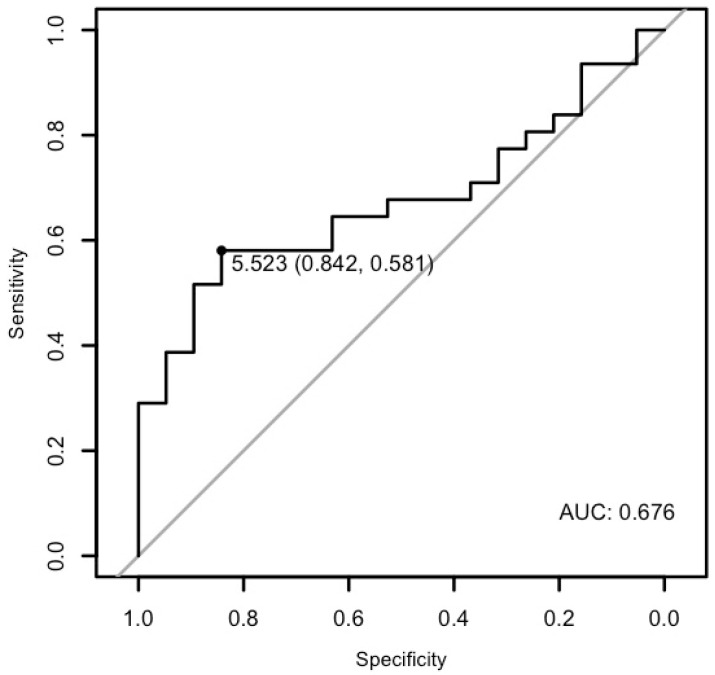
The ROC curve of miR-26b-5p’s predictive value in arrhythmia.

The analysis demonstrated that miR-26b-5p may differentiate patients with cardiac disease from the healthy ones with a specificity of 89.47%, a sensitivity of 53.65%, and an AUC of 0.6944 (*p* < 0.01; Figure 8). Moreover, it may differentiate patients with ventricular arrhythmia from the healthy control with a specificity of 84.21% and a sensitivity of 52.94%, and an AUC of 0.6749 (*p* = 0.033), as well as patients with arrhythmia in general from the healthy control with a specificity of 84.21%, a sensitivity of 58.06%, and an AUC of 0.6757 (*p* = 0.01) (Figure 9 and Figure 10).

**Figure 11:**
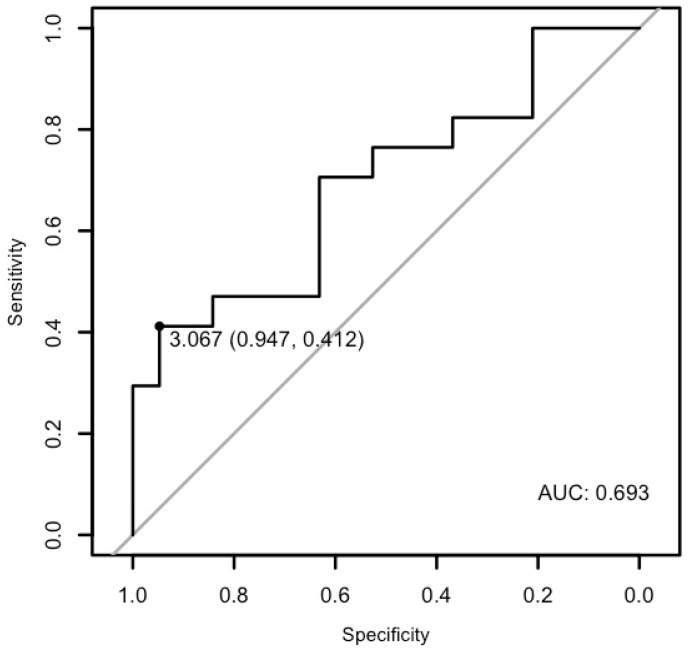
The ROC curve of miR-21-5p’s predictive value in ventricular arrhythmia.

Moreover, the analysis demonstrated that miR-21-5p may differentiate patients with ventricular arrhythmia from healthy controls with a specificity of 94.73% and a sensitivity of 41.17%, and an AUC of 0.6934 (*p* = 0.016, Figure 11).

### 3.4. Multivariable Logistic Regression Analysis of miRNA Expression

A one-unit increase in ΔCt miR-21-5p was associated with a 31.88% increase in the odds of arrhythmia in multivariable analysis, while a one-unit increase in ΔCt miR-26b-5p corresponded to 28.07% increase in univariate and a 36.2% increase in multivariable (Table 8). Moreover, multivariable analysis adjusted for age and sex analysis demonstrated that miR-21-5p and miR-26b-5p were significantly associated with ventricular arrhythmias. A one-unit increase in ΔCt miR-21-5p and miR-26b-5p was associated with a 55.25% and 44.09% increase in the odds of disease, respectively (Table 9). No statistically significant associations were observed in supraventricular arrhythmia, myocarditis, and cardiomyopathy (Table 10, Table 11 and Table 12).

## 4. Discussion

In this study, we investigated miR-1-3p, miR-let-7b-5p, miR-21-5p, and miR-26b-5p levels in children with cardiovascular disease. Our results revealed that miR26-5p is downregulated in young patients with cardiac disease in comparison to healthy individuals. Moreover, miR-26b-5p may also differentiate patients with ventricular arrhythmias as well as arrhythmias in general from healthy individuals. miR-21-5p may also have the potential to differentiate children with ventricular arrhythmia from healthy ones. This may shed new light and perspective on the role of those small molecules in children, as it is an innovative, unexplored topic, with only a few publications discussing it.

### 4.1. Arrhythmias

Arrhythmias constitute a common problem in children with heart disease. We can distinguish between supraventricular and ventricular arrhythmias. The significance of miRNA in arrhythmias in children is not yet fully understood.

miR-1 is abundantly expressed in the heart muscle as well as in the coronary arterial smooth muscles. Moreover it has been confirmed that it is involved in regulating the expression of genes encoding ion channels (such as SCN5A, CACNA1C, KCND2, KCNA5 and KCNE1 KCNQ1, HCN2 and HCN4, KCNJ2) that are known for ensuring the proper conduction of electrical impulses in the heart muscle and thus potentially contributing to regulation of cardiac conduction, repolarization, autorhythmicity and cardiomyocyte calcium handling [9,10,11,12,13,14]. Disturbance in any of those may lead to the development of both ventricular and supraventricular arrhythmias [15].

Sun et al. first reported that plasma miR-1 levels were lower in patients with arrhythmia and SVT than in healthy controls [16]. Contrary findings were reported by Janiszewska et al., who demonstrated that miR-1 expression was significantly higher in the supraventricular arrhythmia group than in the controls [17]. However, in our study, we did not find significant changes in the expression of miR-1 between patients with SVT and healthy individuals. Furthermore, Janiszewska et al. did not find statistical significance in the expression of miR-1 between patients with ventricular arrhythmia and healthy individuals, which supports our findings [17].

Similar to miR-1, let-7b-5p is expressed in vascular smooth muscle cells, endothelial cells, cardiomyocytes, and coronary arterial smooth muscle cells. The let-7 family is also known to be involved in ion channels such as SCN5A, GJC1, GJA1, and CACNA1C expression regulation [18,19,20]. Dysfunctions of those are related to ventricular arrhythmias, AF occurrence, and sudden cardiac death [21,22,23]. As far as researchers know, expression of let-7b-5p in young patients with SVT or VT has not yet been evaluated. In this study, we did not find a statistical difference in the expression of miR-let-7b-5p in patients with SVEx or VEx compared to healthy individuals.

miR-21 is commonly expressed in the human heart. In adults, miR-21 was described as related to atrial fibrillation occurrence [24,25,26,27]. miR-21-5p is involved in the regulation of the Kv1.1 channel (KCNA1 gene expression), which is related to ventricular arrhythmia occurrence; a direct link has not yet been proven [28,29]. We are the first to report that miR-21-5p is downregulated in children with ventricular arrhythmia. Moreover, it may have diagnostic potential; however, it exhibited high specificity but relatively low sensitivity.

On the other hand, miR-26b-5p is known to affect the differentiation of human endometrium-derived mesenchymal stem cells (hEMSCs) into cardiomyocytes, but its influence on arrhythmias in children is limited [30]. In adults, miR-26 is known to regulate potassium channel gene expression (KCNJ2), therefore might be involved in AF occurrence [31,32,33]. In our study, miR-26b-5p was downregulated in patients with cardiac disease in general, in patients with ventricular arrhythmia, and in patients with arrhythmia in general compared to healthy controls. Similarly to miR-21-5p, as a potential biomarker, it exhibited high specificity but relatively low sensitivity; therefore, it cannot yet be used as a reliable indicator of these diseases. We have not found statistical significance of miR-26b-5p expression between patients strictly with supraventricular arrhythmia in comparison with controls.

### 4.2. Myocarditis

Myocarditis is defined as an inflammation of the heart muscle, often caused by viral infection, e.g., Coxsackievirus B (CVB), parvovirus B19, a bacterial agent, or systemic disease.

In children with rheumatic carditis, Gumus et al. did not find a significant difference in miR-1 nor miR-21-5p expression between patients with myocarditis and healthy controls [34], which supports our findings. However, in our study, myocarditis was caused by a viral agent (human herpesvirus HHV-7, which could also be an infection manifesting itself in conjunction with another viral infection, undetected in the test).

On the other hand, Wang et al. investigated pediatric patients who developed viral myocarditis due to CVB, respiratory syncytial virus (RSV), parvovirus B19, HHVs, or other viral pathogens. In these, circulating miR-1 was markedly lower than in healthy controls [35]. Others demonstrated that in adult patients with myocarditis, miR-1 and miR-21 expression is higher than that in healthy controls, whereas expression of miR-26b is lower [36]. Moreover, some studies are showing the influence of miR-26b on inflammatory responses beyond myocarditis [37,38]. However, we are first to report that in children with myocarditis, there has been no significant change in miR-26b-5p’s expression between children with this cardiac condition and healthy ones.

Data on the role of miR-let-7b-5p in myocarditis is limited. In mouse models, miR-let-7b was upregulated in female mice with CVB3 myocarditis [39]. Other studies have shown that the miR-let-7 family is involved in the modulation of viral replication, the host antiviral response, reduction in the innate immune response, and inflammation after infection [40,41,42]. However, there is no data on the expression of miR-let-7b-5p in children with myocarditis so far. We did not find statistically significant changes in the expression of this miRNA between children with myocarditis and healthy controls. However, it must be emphasized that the cohort size for myocarditis was small, as it reflects the low incidence and rarity of these acute pediatric presentations, and further studies should be conducted.

### 4.3. Cardiomyopathies

Cardiomyopathies constitute a broad group of diseases that include hypertrophic cardiomyopathy, dilated cardiomyopathy, and arrhythmogenic right ventricular cardiomyopathy. The underlying causes of these diseases are often environmental (toxins, viruses, inflammation), but also genetic. There is very limited data on miR-1-3p, miR-let-7b-5p, miR-21-5p, and miR-26b-5p in patients with cardiomyopathies. For the study, we selected patients with one of the most common types of cardiomyopathy—hypertrophic cardiomyopathy.

Cardiac hypertrophy associated with congenital heart disease has been linked to elevated levels of miR-1 as well as miR-21 [43]. Others claim that miR-1 may inhibit cardiac hypertrophy and even distinguish between end-stage HCM, dilated cardiomyopathy, and left ventricular dilatation [44,45]. Also, the let-7 family was found to exhibit antihypertrophic properties, and let-7b-5p itself regulated IGFBP3 and JAK2, seen as biomarkers of HCM [46,47]. Overexpression of miR-26 may attenuate cardiac hypertrophy [31]. Together with miR-let-7, miR-26 was upregulated in the model of ventricular hypertrophy in hypertensive patients [48]. In our study, there was no statistical significance in changes in the expression of any of these miRNAs between children with hypertrophic cardiomyopathy and healthy controls. However, similarly to the myocarditis cohort, the group was small and further studies should be conducted.

### 4.4. Impact of Age and Sex

Several studies have shown differences in miRNA expression in children of different ages as well as in adults. miR-1 was expressed in adulthood only and was not associated with aging, while let-7a was constantly expressed from infancy and childhood, up-regulated in young adulthood, and then showed expression diminishing with aging [49]. Let-7e exhibited higher expression in older children compared to newborns [50]. miR-21 and miR-26 also seem to be age-associated. Moreover, sex may influence the expression of some miRNAs [51,52]. In our population miR-21-5p and miR-26b-5p were significantly associated with ventricular arrhythmia and arrhythmia in general independently of age and sex. However, it should be noted that our study group included children aged 10–18, which may affect the results; younger children were not included (the average age in the population was 13.79 years). Moreover, males accounted for 57% of the population, which may slightly affect the results.

## 5. Limitations

The authors are aware of the limitations of this study. Relatively small groups of children with myocarditis and cardiomyopathies were included. The concentrations of miRNA molecules may have been influenced by undiagnosed, asymptomatic medical conditions or allergies that have not yet been identified, as well. Moreover, children from the control group—however, without structural heart disease—cannot be considered strictly healthy volunteers. Potential underlying physiological differences between these individuals and the general pediatric population may influence the results. In the future, we intend to expand the research group to assess the impact of chosen miRNAs on children with CVDs and explore their potential use as new biomarkers.

## 6. Conclusions

miR-26b-5p may distinguish young patients with CVDs and those with ventricular arrhythmia and arrhythmia in general from healthy individuals. miR-21-5p may also be seen as a potential biomarker of ventricular arrhythmia in children. In contrast, miR-1-3p and miR-let-7b-5p may not participate in the development or attenuation of CVDs in children. Further studies involving a larger sample size are required to obtain sufficient data and validate these findings.

## Figures and Tables

**Table 1:** Study population characteristics and subdivision.

Variables	Total n = 63	Control n = 20	Cardiac Disease n = 43	Arrhythmia n = 33	Supraventricular Arrhythmia n = 17	Ventricular Arrhythmia n = 18	Myocarditis n = 7	Cardiomyopathies n = 4
Average age [years]	13.79	13.75	13.81	13.58	13.22	13.88	15.26	14.0
Sex distribution [% of males]	57	50	60	55	55	53	86	75
CK [IU/L]	82 ± 44	77.5 ± 39.25	83 ± 41	83 ± 34	92 ± 36	77 ± 28	61 ± 116	156.5 ± 69.75
CK-MB [IU/L]	16 ± 6.75	15 ± 5	17 ± 7	17 ± 7	18 ± 6	17 ± 7	16 ± 12.5	17.5 ± 5.5
Troponin [ng/L]	4.25 ± 3.08	3.7 ± 2.7	5.1 ± 11.8	4.6 ± 2.5	5.85 ± 9.13	4.2 ± 1.7	171 ± 1079.8	9.45 ± 272.7

CK—creatine kinase; CK-MB—creatine kinase MB.

**Table 2:** The comparison of ΔCt values between patients with and without cardiac diseases.

Variables	Cardiac Disease n = 43	Control n = 20	*p* Value
miR-1-3p	9.66 ± 2.88	8.78 ± 3.15	0.3950
miR-let-7b-5p	2.92 ± 3.05	2.30 ± 1.61	0.1661
miR-21-5p	2.28 ± 3.14	1.36 ± 1.77	0.0541
miR-26b-5p	5.71 ± 3.60	4.44 ± 2.30	0.0155

**Table 3:** The comparison of ΔCt values between patients with and without supraventricular arrhythmias.

Variables	Supraventricular Arrhythmia n = 17	Control n = 20	*p* Value
miR-1-3p	7.92 ± 3.36	8.78 ± 3.15	0.7741
miR-let-7b-5p	2.68 ± 3.88	2.30 ± 1.61	0.3275
miR-21-5p	1.20 ± 4.83	1.36 ± 1.77	0.6532
miR-26b-5p	5.71 ± 5.14	4.44 ± 2.30	0.1017

**Table 4:** The comparison of ΔCt values between patients with and without ventricular arrhythmias.

Variables	Ventricular Arrhythmia n = 18	Control n = 20	*p* Value
miR-1-3p	9.48 ± 3.00	8.78 ± 3.15	0.2524
miR-let-7b-5p	2.92 ± 2.38	2.30 ± 1.61	0.4146
miR-21-5p	2.18 ± 3.50	1.36 ± 1.77	0.0486
miR-26b-5p	5.62 ± 3.60	4.44 ± 2.30	0.0042

**Table 5:** The comparison of ΔCt values between patients with and without arrhythmias.

Variables	Arrhythmia n = 33	Control n = 20	*p* Value
miR-1-3p	8.67 ± 3.03	8.78 ± 3.15	0.6896
miR-let-7b-5p	2.88 ± 3.57	2.30 ± 1.61	0.3170
miR-21-5p	2.17 ± 4.36	1.36 ± 1.77	0.1354
miR-26b-5p	5.71 ± 3.99	4.44 ± 2.30	0.0387

**Table 6:** The comparison of ΔCt values between patients with and without myocarditis.

Variables	Myocarditis n = 7	Control n = 20	*p* Value
miR-1-3p	10.19 ± 1.55	8.78 ± 3.15	0.1427
miR-let-7b-5p	2.55 ± 2.27	2.30 ± 1.61	0.3058
miR-21-5p	2.57 ± 1.38	1.36 ± 1.77	0.0664
miR-26b-5p	4.74 ± 2.28	4.44 ± 2.30	0.1006

**Table 7:** The comparison of ΔCt values between patients with and without cardiomyopathies.

Variables	Cardiomyopathies n = 4	Control n = 20	*p* Value
miR-1-3p	9.30 ± 2.69	8.78 ± 3.15	0.5290
miR-let-7b-5p	3.55 ± 0.95	2.30 ± 1.61	0.0811
miR-21-5p	2.58 ± 2.15	1.36 ± 1.77	0.1354
miR-26b-5p	6.14 ± 1.59	4.44 ± 2.30	0.0620

**Table 8:** Multivariable logistic regression analysis of miRNA expression in arrhythmia with adjustment for age and sex.

Arrhythmia	OR	LOWER—95%	UPPER—95%	Std. Error	z Value	*p* Value
Univariate ΔCt miR-1-3p	1.0534	0.8161	1.3702	0.1297	0.4009	0.6885
Multivariable						
ΔCt miR-1-3p	1.0842	0.8317	1.4308	0.1359	0.5946	0.5521
Age [y.o.]	1.9531	0.5377	7.5594	0.6663	1.0046	0.3151
Male	1.0724	0.7604	1.5257	0.1751	0.3992	0.6897
Univariate ΔCt miR-let-7b-5p	1.1699	0.9686	1.4957	0.1080	1.4534	0.1461
Multivariable						
ΔCt miR-let-7b-5p	1.2012	0.9754	1.5807	0.1196	1.5327	0.1253
Age [y.o.]	1.6624	0.5001	5.7979	0.6189	0.8212	0.4115
Male	1.0533	0.7569	1.4799	0.1685	0.3078	0.7582
Univariate ΔCt miR-21-5p	1.2491	1.0207	1.6660	0.1230	1.8090	0.0704
Multivariable						
ΔCt miR-21-5p	1.3188	1.0509	1.8135	0.1378	2.0081	0.0446
Age [y.o.]	2.3512	0.6301	9.7348	0.6902	1.2387	0.2155
Male	1.1098	0.7852	1.5883	0.1769	0.5888	0.5560
Univariate ΔCt miR-26b-5p	1.2807	1.0411	1.6807	0.1203	2.0557	0.0398
Multivariable						
ΔCt miR-26b-5p	1.3620	1.0717	1.8623	0.1404	2.2002	0.0278
Age [y.o.]	2.3305	0.6333	9.6809	0.6858	1.2338	0.2173
Male	1.1449	0.8073	1.6517	0.1797	0.7529	0.4515

**Table 9:** Multivariable logistic regression analysis of miRNA expression in ventricular arrhythmia with adjustment for age and sex.

Ventricular Arrhythmia	OR	LOWER—95%	UPPER—95%	Std. Error	z Value	*p* Value
Univariate ΔCt miR-1-3p	1.2163	0.8918	1.7235	0.1641	1.1928	0.2329
Multivariable						
ΔCt miR-1-3p	0.9191	1.8864	0.1790	1.3930	0.1636	0.9191
Age [y.o.]	0.4464	11.2731	0.8092	0.9190	0.3581	0.4464
Male	0.7585	1.7165	0.2038	0.5961	0.5511	0.7585
Univariate ΔCt miR-let-7b-5p	0.9020	1.6019	0.1390	1.0647	0.2870	0.9020
Multivariable						
ΔCt mir7	1.1694	0.9054	1.6440	0.1443	1.0845	0.2782
Age [y.o.]	1.3667	0.3378	5.7723	0.7145	0.4373	0.6619
Male	1.0734	0.7395	1.5732	0.1888	0.3750	0.7077
Univariate ΔCt miR-21-5p	1.4118	1.0666	2.1960	0.1837	1.8768	0.0605
Multivariable						
ΔCt miR-21-5p	1.5525	1.1066	2.5830	0.2188	2.0104	0.0444
Age [y.o.]	2.7527	0.5466	16.8737	0.8582	1.1799	0.2380
Male	1.1319	0.7598	1.7274	0.2047	0.6052	0.5450
Univariate ΔCt miR-26b-5p	1.3481	1.0373	1.9511	0.1581	1.8891	0.0589
Multivariable						
ΔCt miR-26b-5p	1.4409	1.0658	2.1885	0.1831	1.9951	0.0460
Age [y.o.]	2.1869	0.4605	12.2928	0.8228	0.9510	0.3416
Male	1.1595	0.7769	1.7830	0.2070	0.7149	0.4747

**Table 10:** Multivariable logistic regression analysis of miRNA expression in supraventricular arrhythmia with adjustment for age and sex.

Supraventricular Arrhythmia	OR	LOWER—95%	UPPER—95%	Std. Error	z Value	*p* Value
Univariate ΔCt miR-1-3p	0.9483	0.6825	1.3017	0.1605	−0.3309	0.7408
Multivariable						
ΔCt miR-1-3p	0.9593	0.6817	1.3397	0.1678	−0.2476	0.8044
Age [y.o.]	1.9538	0.4064	10.4743	0.8130	0.8238	0.4100
Male	1.017	0.6552	1.5858	0.2203	0.0787	0.9373
Univariate ΔCt miR-let-7b-5p	1.2466	0.9891	1.7480	0.1406	1.5683	0.1168
Multivariable						
ΔCt miR-let-7b-5p	1.3235	0.9854	2.0303	0.1778	1.5765	0.1149
Age [y.o.]	1.9881	0.4840	8.9845	0.7352	0.9347	0.3500
Male	1.0943	0.7139	1.7068	0.2183	0.4126	0.6799
Univariate ΔCt miR-21-5p	1.1850	0.9319	1.6143	0.1344	1.2631	0.2065
Multivariable						
ΔCt miR-21-5p	1.2501	0.9538	1.7651	0.15034	1.4847	0.1376
Age [y.o.]	2.1488	0.4703	11.3731	0.7979	0.9587	0.3377
Male	1.1221	0.7400	1.7443	0.2143	0.5378	0.5907
ΔCt miR-26b-5p	1.2738	1.0010	1.7417	0.1346	1.7979	0.0722
Multivariable						
Univariate ΔCt miR-26b-5p	1.3715	1.0335	2.0256	0.1695	1.8633	0.0624
Age [y.o.]	2.4904	0.5554	13.4209	0.7951	1.1475	0.2512
Male	1.1750	0.7705	1.8407	0.2178	0.7406	0.4589

**Table 11:** Multivariable logistic regression analysis of miRNA expression in myocarditis with adjustment for age and sex.

Myocarditis	OR	LOWER—95%	UPPER—95%	Std. Error	z Value	*p* Value
Univariate ΔCt miR-1-3p	1.3906	0.8706	2.4687	0.2573	1.2813	0.2001
Multivariable						
ΔCt miR-1-3p	1.5069	0.8216	3.3751	0.3439	1.1923	0.2332
Age [y.o.]	15.5089	1.4453	472.8243	1.3943	1.9661	0.0493
Male	1.9554	1.1162	4.3899	0.3306	2.0284	0.0425
Univariate ΔCt miR-let-7b-5p	1.5539	0.9244	3.1104	0.3042	1.4492	0.1473
Multivariable						
ΔCt miR-let-7b-5p	1.4230	0.7911	3.6436	5.1579	−2.4519	0.0142
Age [y.o.]	10.4813	0.9985	294.8006	0.3725	0.9471	0.3436
Male	1.8880	1.0929	4.0662	1.3595	1.7283	0.0839
Univariate ΔCt miR-21-5p	1.8521	0.9767	4.4287	0.3726	1.6540	0.0981
Multivariable						
ΔCt miR-21-5p	2.6927	1.1005	9.9897	0.5442	1.8202	0.0687
Age [y.o.]	29.6429	1.8989	2304.3104	1.6995	1.9943	0.0461
Male	2.1960	1.1845	5.8254	0.3775	2.0840	0.0372
Univariate ΔCt miR-26b-5p	1.5967	0.9509	3.2455	0.3063	1.5278	0.1266
Multivariable						
ΔCt miR-26b-5p	2.1765	1.0309	6.3192	0.4551	1.7088	0.0875
Age [y.o.]	28.7462	1.8910	2052.2450	1.6832	1.9953	0.0460
Male	2.0979	1.1623	5.1773	0.3549	2.0876	0.0368

**Table 12:** Multivariable logistic regression analysis of miRNA expression in cardiomyopathy with adjustment for age and sex.

Cardiomyopathy	OR	LOWER—95%	UPPER—95%	Std. Error	z Value	*p* Value
Univariate ΔCt miR-1-3p	1.1984	0.6902	2.2561	0.2884	0.6274	0.5304
Multivariable						
ΔCt miR-1-3p	1.3435	0.7181	3.0626	0.3488	0.8465	0.3973
Age [y.o.]	5.1154	0.4389	139.4834	1.3678	1.1933	0.2327
Male	1.3200	0.6912	2.7864	0.3357	0.8270	0.4083
Univariate ΔCt miR-let-7b-5p	2.5607	0.9550	11.1743	0.6131	1.5337	0.1251
Multivariable						
ΔCt miR-let-7b-5p	3.720	1.060	30.536	0.802	1.638	0.102
Age [y.o.]	4.359	0.324	150.172	1.453	1.013	0.311
Male	1.553	0.795	3.454	0.356	1.236	0.216
Univariate ΔCt miR-21-5p	1.975	0.893	5.959	0.469	1.452	0.146
Multivariable						
ΔCt miR-21-5p	3.2802	1.0719	22.2799	0.7353	1.6156	0.1062
Age [y.o.]	6.6466	0.4890	223.0427	1.4645	1.2934	0.1959
Male	1.7683	0.8434	4.8411	0.4197	1.3582	0.1744
Univariate ΔCt miR-26b-5p	2.4123	1.0735	8.4402	0.5052	1.7430	0.0813
Multivariable						
ΔCt miR-26b-5p	4.9638	1.3127	109.5118	1.0201	1.5706	0.1163
Age [y.o.]	5.3069	0.3257	203.7710	1.5270	1.0930	0.2744
Male	2.1134	0.9084	9.5453	0.5332	1.4034	0.1605

## Data Availability

The data presented in this study are available on request from the corresponding author.
